# Psymberin, a marine-derived natural product, induces cancer cell growth arrest and protein translation inhibition

**DOI:** 10.3389/fmed.2022.999004

**Published:** 2022-12-20

**Authors:** Divya L. Dayanidhi, Jason A. Somarelli, John B. Mantyh, Gabrielle Rupprecht, Roham Salman Roghani, Sophia Vincoff, Iljin Shin, Yiquan Zhao, So Young Kim, Shannon McCall, Jiyong Hong, David S. Hsu

**Affiliations:** ^1^Division of Medical Oncology, Department of Medicine, Duke University Medical Center, Durham, NC, United States; ^2^Center for Genomics and Computational Biology, Duke University, Durham, NC, United States; ^3^Department of Chemistry, Duke University, Durham, NC, United States; ^4^Department of Molecular Genetics and Microbiology, Duke University, Durham, NC, United States; ^5^Department of Pathology, Duke University, Durham, NC, United States; ^6^Department of Pharmacology and Cancer Biology, Duke University School of Medicine, Durham, NC, United States

**Keywords:** patient-derived organoids, patient-derived models of cancer, precision medicine, psymberin, high-throughput screening, protein translation

## Abstract

Colorectal cancer (CRC) is the third most prevalent form of cancer in the United States and results in over 50,000 deaths per year. Treatments for metastatic CRC are limited, and therefore there is an unmet clinical need for more effective therapies. In our prior work, we coupled high-throughput chemical screens with patient-derived models of cancer to identify new potential therapeutic targets for CRC. However, this pipeline is limited by (1) the use of cell lines that do not appropriately recapitulate the tumor microenvironment, and (2) the use of patient-derived xenografts (PDXs), which are time-consuming and costly for validation of drug efficacy. To overcome these limitations, we have turned to patient-derived organoids. Organoids are increasingly being accepted as a “standard” preclinical model that recapitulates tumor microenvironment cross-talk in a rapid, cost-effective platform. In the present work, we employed a library of natural products, intermediates, and drug-like compounds for which full synthesis has been demonstrated. Using this compound library, we performed a high-throughput screen on multiple low-passage cancer cell lines to identify potential treatments. The top candidate, psymberin, was further validated, with a focus on CRC cell lines and organoids. Mechanistic and genomics analyses pinpointed protein translation inhibition as a mechanism of action of psymberin. These findings suggest the potential of psymberin as a novel therapy for the treatment of CRC.

## Introduction

Colorectal cancer (CRC) is the third most commonly occurring form of cancer in the United States and is the cause of over 50,000 deaths per year (1). At initial diagnosis, approximately 20% of patients will have distant metastasis, and another 25–30% of patients with stage II/III disease will develop metastasis (2). Currently, the use of chemotherapy in the metastatic setting can palliate symptoms and improve survival, but do not result in cures for patients. If left untreated, patients with colorectal metastasis can expect an overall survival of approximately 9 months, but with combination therapy, survival can be improved to greater than 24 months (3, 4). The last two drugs that have been approved by the U.S. Food and Drug Administration for the treatment of refractory CRC were regorafenib (5) in 2014 and lonsurf (6) in 2015. Despite these improvements, there remains a lack of new drugs for the treatment of advanced CRC. Unfortunately, the failure rate for new cancer drugs is more than 80% in Phase II and 50% in Phase III trials (7). As a result, despite our advances, CRC still remains an incurable and debilitating disease, and there is an unmet clinical need to develop new therapeutics for CRC.

In our previous work, we developed a precision medicine pipeline to facilitate the identification and validation of new therapies in CRC and other solid tumors (8–11). These studies highlighted the utility of a precision medicine pipeline to identify, test, and characterize novel therapeutics using patient-matched low passage cell lines and patient-derived xenografts (PDXs). Cell lines provide a rapid and low-cost resource to test thousands of compounds and perform genomics studies, while matched PDXs provide robust *in vivo* models to validate top candidate therapies. Despite the utility of this platform, it is limited by (1) the reliance on the generation of cell lines, which can take months to establish and characterize and do not faithfully recapitulate the tumor microenvironment and (2) the establishment of PDXs, which are costly and often time-consuming to produce and maintain.

**FIGURE 1:**
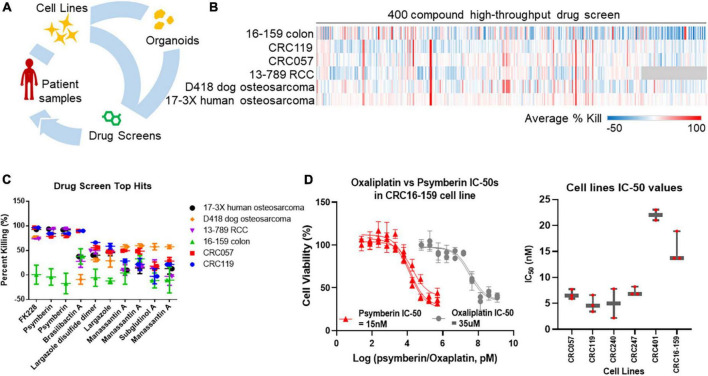
Identification of psymberin as a potential anti-cancer therapeutic agent for colorectal cancer (CRC). **(A)** Tumor samples or low-passage cell lines from patients are used to establish organoid cultures *ex vivo*. These organoids are then used in drug screens. **(B)** A high-throughput screen with 400 compounds in six cell lines. Blue indicates a negative average percent killing (net growth) and red indicates a high average percent killing. Compounds that were not tested for a cell line are shown in gray. **(C)** The top ten hits from the high throughput screen in the same six cell lines. Some compounds were synthesized in multiple batches and were therefore tested more than once. **(D)** Dose-response comparison between oxaliplatin and psymberin in the same CRC cell line (left) and comparison of psymberin IC-50 values across different CRC cell lines (right).

Given the limitations of our current pipeline, we have turned to patient-derived organoids. Patient-derived organoids are increasingly being accepted as a “standard” preclinical model that is both more representative of *in vivo* tumor physiology than cell lines and a low-cost rapid alternative to PDXs (12–14). We have therefore adapted our precision medicine pipeline to incorporate the use of patient-derived organoids (Figure 1A). Using this new pipeline, we first performed a high-throughput drug screen using a compound library on a panel of early-passage cell lines from multiple solid tumors to identify potential therapeutic agents. From this screen, we identified psymberin as one of the top small molecules with potent growth inhibition activity.

Psymberin, also known as irciniastatin A, belongs to a group of biologically active natural products called polyketides. Psymberin was independently discovered in 2004 by the research groups of Crews and Pettit from the sponges *Psammocinia sp*. and *Ircinia ramose*, respectively, and it has been a marine natural product of immense interest since its isolation (15–18). Psymberin has been tested against 60 cancer cell lines and displayed potent cytotoxicity against melanoma, breast, and colon cancer cell lines (LC_50_ < 2.5 × 10^–9^ M). While these studies pinpoint psymberin as an effective anti-cancer agent, its molecular mechanisms are not extensively understood.

Identification of psymberin as a top hit in our screens prompted us to further characterize the activity and mechanisms of action for psymberin. RNA-Seq on cells treated with psymberin identified negative enrichment of protein translation as a potential mechanism of action. The impact on translation was verified using a fluorescence-based assay of translation inhibition. The rapid inhibition of protein translation within hours was concomitant with the activation of p38, a stress response pathway, and cell cycle arrest. Together, our results pinpoint psymberin as a potent protein translation inhibitor with anti-cancer properties in CRC.

## Materials and methods

### Establishment and maintenance of low-passage cell lines and organoids

Colorectal cancer patient tissue samples were collected under a Duke Institutional Review Board approved protocol (Pro00089222), obtained from the National Cancer Institute’s Cooperative Human Tissue Network or obtained through Duke University BioRepository and Precision Pathology Center. CRC cell lines were established from patient tissue samples as previously described (8). Briefly, patient samples were processed and injected into SCID beige mice to grow as PDXs. After the tumors grew to ∼0.5 cm^3^, tumors were then harvested, homogenized, and grown in tissue-culture treated dishes, with subsequent clonal selection as indicated (8). Low-passage osteosarcoma lines were generated as described previously (9). All cell lines were maintained in Dulbecco’s Modified Eagle Medium (DMEM) media supplemented with 10% fetal bovine serum (FBS) and 1% penicillin/streptomycin.

To establish organoids from existing cell lines, 2 × 10^6^ cells from each line were subcutaneously injected into SCID beige mice. After the tumors grew to ∼0.5 cm^3^, mice were euthanized following Duke Institutional Animal Care and Use Committee (IACUC)-approved protocols, and the tumor was then harvested. Tumors were mechanically digested in C-tubes with 10 mL of DMEM using a gentleMACS Dissociator (Miltenyi Biotec) and running the m_impTumor_01.01 protocol twice. Cells and tissue fragments were filtered through 70 μm filters and centrifuged at 500 *g* for 5 min. The supernatant was aspirated. 1.25 × 10^5^ cells were plated in 50 μL domes composed of 30% cell suspension in media and 70% Matrigel (Corning). CRC organoids were maintained in CRC media, which consisted of DMEM F12 media supplemented with 10 mM HEPES, 1X Glutamax, 100 μ/mL Penicillin/Streptomycin, 500 nM A83-01, 1X B27 without vitamin A, 50 ng/mL EGF, 10 nM Gastrin-1, 1.25 mM N-Acetylcysteine, 10 mM Nicotinamide, 100 ng/mL Noggin, 100 μg/mL Primocin, 10 nM Prostaglandin E2, 100 ng/mL R-Spondin 1, and 3 μM SB20210.

All cell lines and organoids were maintained at 37°C in a humidified incubator at 5% CO_2_.

### High-throughput drug screen

Cells from six early passage cell lines (16–159 colon, CRC119, CRC057, 13–789 RCC, D418 canine osteosarcoma, and 17-3X human osteosarcoma) were provided to the Duke Functional Genomics Core Facility for testing with a compound library of 400 natural products, intermediates, and drug-like compounds. A subset of compounds was synthesized in multiple batches and were therefore tested more than once to ensure reproducibility across different batches. The high-throughput screen was performed as previously described (8). Briefly, 384-well plates were stamped with each of the compounds from the library at a final concentration of 1 μM. Cells from each of the lines were plated in these pre-stamped plates at a density of 1 × 10^3^ cells/well. Cell viability was assessed using the Cell Titer-Glo luminescent Cell Viability Assay kit (Promega, Madison, WI, USA) after 72 h. Percent killing was calculated as follows: 100*[1–(average Cell Titer-Glo_*drug*_/average Cell Titer-Glo_*DMSO*_)].

### Cell line drug sensitivity assays

Does response curves for psymberin and oxaliplatin were performed in the following six cell lines: CRC057, CRC119, CRC240, CRC247, CRC401, and CRC16-159. Stock solutions at 10 μM for psymberin and oxaliplatin were made in DMSO and phosphate buffered saline (PBS), respectively. Once cells were 70% confluent, they were plated into 96-well plates at a concentration of 4 × 10^3^ cells per well and incubated for 24 h. Cells were then treated with a series of 10 different concentrations in media (DMEM + 10% FBS + 1% penicillin/streptomycin) of psymberin or oxaliplatin starting from 1 μM and 300 μM, respectively, with a serial dilution factor of three. Five replicates were performed for each drug concentration. After incubation in the presence of the drug for 2 days, cell viability was evaluated using the Cell Titer-Glo luminescent Cell Viability Assay kit (Promega, Madison, WI, USA). All drug sensitivity assays were performed in triplicate. Half maximum inhibitory concentration (IC_50_) values were calculated using a non-linear curve fit with the log(inhibitor) vs. response (3 parameters) function in GraphPad Prism (La Jolla, CA, USA).

### Organoid drug sensitivity assays

Organoids were grown in 24 well plates at 37°C for approximately 3 days, after which they were re-plated in 96-well plates for drug sensitivity assays. To do this, media was aspirated from the wells and 1 mL of PBS was added to each well to detach the Matrigel domes from the bottom of the wells. After collecting the Matrigel, wells were washed again with 500 μL of PBS to collect any remaining Matrigel. The Matrigel and PBS was centrifuged for 7 min at 400 × *g*. Supernatants were removed and 500 μL of Trypsin was added to each tube and incubated for 2 min to dissolve the Matrigel. Trypsin was neutralized by the addition of DMEM with 10% FBS and the whole contents were centrifuged for another 3 min at 400 × *g*. Pellets were collected and cells were counted after resuspension in CRC media. Cells were then mixed with Matrigel in a 1:1 ratio and 5 μL of mixture containing 2 × 10^3^ cells was added to the center of each well in a 96-well plate. The 96 well plates were incubated for 10–15 min to allow the Matrigel to solidify before adding 50 μL of CRC media to each well and incubating at 37°C for 72 h. Organoids were treated with a series of six different concentrations of psymberin starting from 1 μM with a dilution factor of five. After incubation in the presence of the drug for 2 days, cell viability was quantified *via* the Cell Titer-Glo luminescent Cell Viability Assay (Promega, Madison, WI, USA). All drug sensitivity assays were performed in triplicate. IC_50_ values were calculated using a non-linear curve fit with the log(inhibitor) vs. response (3 parameters) function in GraphPad Prism (La Jolla, CA, USA).

### Protein synthesis assays

Nascent protein synthesis was quantified in the CRC119 cell line using Click-iT^®^ HPG Alexa Fluor^®^ 488 Protein Synthesis Assay Kit (Thermo Fisher Scientific). CRC119 cells were plated into 96-well plates at the concentration of 4 × 10^3^ cells/well in drug-free medium and allowed to recover overnight before treating them with either 1% DMSO, 3X psymberin IC_50_, or 50 μM cycloheximide. Drug-containing medium was removed at 1 and 6 h intervals, and medium containing 50 μM l-homopropargylglycine (Click-iT^®^ HPG) was added in the dark. After incubation for 30 min, medium containing Click-iT^®^ HPG was removed, and cells were washed once with PBS. Cells were fixed in 3.7% formaldehyde and permeabilized with 0.5% Triton^®^ X-100 in PBS. HPG incorporation was detected using the Click-iT^®^ reaction cocktail prepared according to the vendor’s guidelines. Plates were incubated for 30 min at room temperature followed by washing wells with ClickiT^®^ reaction rinse buffer and PBS. Plates were imaged using an Incucyte^®^ S3 live cell imaging system. Luminescence was quantified in FIJI/ImageJ.

### Protein isolation and western blotting

CRC119 cells were treated with psymberin at 3X their IC_50_ at different time points. Cells were lysed in radio-immunoprecipitation assay buffer supplemented with protease and phosphatase inhibitors (Thermo Fisher Scientific). Protein concentration was assessed by using the BCA Protein Assay (Bio-Rad). A total of 60 μg of total protein from each sample was electrophoretically separated on 4–20% sodium dodecyl sulfate polyacrylamide gels (Bio-Rad, USA) and transferred to polyvinylidene difluoride (PVDF) membranes (Bio-Rad). After blocking the membranes with blocking buffer (Bio-Rad), membranes were incubated overnight with primary antibodies for phospho-p38, p38, poly (ADP-ribose) polymerase (PARP), or cleaved PARP. Glyceraldehyde 3-phosphate dehydrogenase (GAPDH) was used as a loading control. Appropriate secondary antibodies were added subsequently. Blots were scanned using a Licor Odyssey imaging system.

### Cell cycle analysis

CRC119 and CRC16-159 cells were seeded at 3 × 10^5^ cells per well in 6-well plates and incubated until they reached 60% confluence. Cells were treated with either 3X their IC_50_ of psymberin or 0.1% DMSO. After 24 and 48 h, cells were harvested and washed two times with PBS followed by fixing in 80% ethanol for 30 min. Subsequently, cells were washed twice more with PBS and resuspended in cell staining buffer (0.1% Triton X-100, 0.1 mM EDTA disodium, 50 μg/mL RNAse A, and 50 μg/mL PI in PBS) immediately prior to flow cytometry. Flow cytometry-based cell cycle analysis was performed by the Duke University Flow Cytometry Shared Resource. A Chi-square test was used to estimate statistical reliability of the observations.

### RNA-seq

A total of 8 × 10^4^ CRC119 and 16–159 cells were plated in 6-well plates and allowed to incubate overnight. Cells were then treated with 3X their IC_50_ of psymberin and RNA was extracted after 16 h using the RNEasy Mini Kit (Qiagen). RNA-Seq data was processed using the TrimGalore toolkit (19) which employs Cutadapt (20) to trim low-quality bases and Illumina sequencing adapters from the 3′ end of the reads. Only reads that were 20 nt or longer after trimming were kept for further analysis. Reads were mapped to the GRCh37v75 version of the human genome and transcriptome (21) using the STAR RNA-seq alignment tool (22). Reads were kept for subsequent analysis if they mapped to a single genomic location. Gene counts were compiled using the (23) tool (“HTSeq: High-throughput sequence analysis in Python”). Only genes that had at least 10 reads in any given library were used in subsequent analysis. Normalization and differential expression was carried out using the DESeq2 (24) Bioconductor (25) package with the R statistical programming environment (“The R Project for Statistical Computing”) (26). The false discovery rate was calculated to control for multiple hypothesis testing. Gene set enrichment analysis (27) was performed to identify differentially regulated pathways and gene ontology terms for each of the comparisons performed. False discovery rate cutoffs for positive and negative enrichment were <0.25 and <0.15, respectively. Normalized enrichment score cutoffs for positive and negative enrichment were >0 and <−1.8, respectively. Upset plots and heatmaps were constructed in R using the ComplexUpset and ComplexHeatmap packages.

### Determination of apoptosis using the IncuCyte^®^ annexin V green reagent

The IncuCyte^®^ Annexin V Green Reagent (Sartorius), which is a highly-selective phosphatidylserine (PS) cyanine fluorescent dyes that enables real-time evaluation and quantification of cell death, was used. CRC 119 and CRC 16–159 cell lines were seeded into 96-well plates at the concentration of 4,000 cell/well in drug free medium and incubated for 24 h to allow for attachment. Cells were treated with either Psymberin or Cisplatin (as a positive control for inducing apoptosis in CRC) at 3X their IC_50_ dose. Annexin V Green Reagent was added to the wells at the same time according to manufacturer’s protocol. An automated platform (Incucyte^®^) was used for imaging plates at the beginning and 24 h after the treatment. PS exposure on the extracellular surface following apoptosis, enables binding of the IncuCyte Annexin V Reagent resulting in a bright and photostable fluorescent signal.

### Drug sensitivity assays using MicroOrganoSpheres

CRC404 and CRC420 organoids were grown in 50 μL Matrigel domes in CRC media at 37°C in a humidified incubator at 5% CO_2_. Once the organoids were confluent, the media was aspirated from the wells and 1 mL of PBS was added to each well to detach the Matrigel dome from the bottom of the well. The Matrigel was centrifuged at 750 *g* for 5 min. Matrigel was dissolved, organoids were broken down using 1 mL of TrypLE Express (Gibco) and the mixtures were incubated for 5 min. TrypLE was neutralized by adding 5 mL of DMEM F12 media with 10% FBS and 1% penicillin/streptomycin. After centrifuging at 750 *g* for 5 min, the media was aspirated. Organoid cell suspensions were used to make MicroOrganoSpheres as previously described (28).

Stock solutions for psymberin and two analogs (psy-064 and psy-076) were made at 1 mM in DMSO. MicroOrganoSpheres were plated in 96-well plates at a concentration of 100 MicroOrganoSpheres/well with 1X of each component in the RealTime Glo MT Cell Viability Assay kit (RTG; Promega, Madison, WI, USA). MicroOrganoSpheres were treated with each of the three compounds in a nine-point dilution series starting from 1 μM with a dilution factor of three and five replicates per dose. Fluorescence was measured every day for 3 days using a Varioskan Lux plate reader (Thermo Fisher Scientific). IC_50_ values were calculated using a non-linear curve fit with the log(inhibitor) vs. response (3 parameters) function in GraphPad Prism (La Jolla, CA, USA).

## Results

### A high-throughput natural product screen identifies psymberin as a potential anti-cancer compound

To identify potential anti-cancer therapeutic agents, we performed a high-throughput drug screen on six early passage cell lines using a library of natural products and drug-like compounds. The six cell lines included three CRC lines (16–159, CRC119, and CRC057) (8), one renal cell carcinoma line (13–789 RCC), and two osteosarcoma lines (D418 canine and 17-3X human) (9). Less than 10% of the compounds were effective across the entire panel of cell lines, with the 17-3X human osteosarcoma cell line the most broadly sensitive to the library and the 16–159 CRC cell line the most broadly resistant (see Figure 1B). Across the cell lines, compounds with an average percent killing above 50% included nine compounds against 16–159 CRC cells, 10 compounds in CRC119 cells, 6 compounds in CRC057 cells, 4 compounds in 13–789 RCC cells, 1 compound in D418 canine osteosarcoma cells, and 3 compounds in 17-3X human osteosarcoma cells. To identify the most effective drugs, we focused on compounds with the highest average percent killing across the entire panel of cell lines. A subset of the compounds was synthesized in different batches and tested more than once to ensure reproducibility across different batches. In these cases, analysis of the screen data identified some compounds more than once as consistent top hits, which provides further support for these hits (Figure 1C). Among these top hits, FK228 and psymberin had an average percent killing ≥50% in all but one cell line (see Figure 1C). FK228, also known as romidepsin, has already been approved for the treatment of lymphoma (29), suggesting our screening strategy is capable of identifying natural products with efficacy as anti-cancer agents. Since FK228 is already approved as an anti-cancer agent, we focused on psymberin for further validation.

To understand the relative potency of psymberin against CRC, we compared dose response curves for psymberin and oxaliplatin, a standard-of-care drug for the treatment of CRC. These analyses indicated that psymberin is over 2,000 times more potent than oxaliplatin, with an IC_50_ of approximately 15 nM (Figure 1D). The low nanomolar IC_50_ values were consistent across six CRC lines, with IC_50_ values below 25 nM for every line and below 10 nM for four of the six lines (Figure 1D and Supplementary Figure 1).

### Psymberin inhibits protein synthesis

**FIGURE 2:**
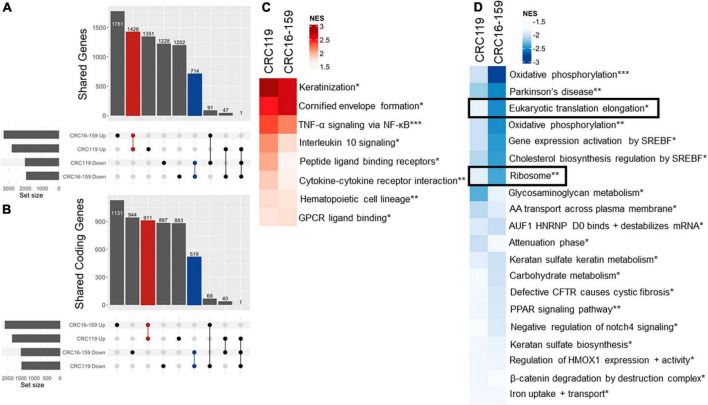
RNA-Seq analysis of psymberin treatment in colorectal cancer (CRC) cell lines. **(A)** Upset plot showing overlap of up- and down-regulated genes after psymberin treatment between two CRC cell lines: CRC119 and CRC16-159. Red indicates the number corresponding to upregulated genes consistent between both lines; blue indicates the number of genes consistently downregulated in both lines. **(B)** Same as 3A with coding genes only. **(C)** Most significant positively enriched pathways after psymberin treatment, determined using gene set enrichment analysis. **(D)** Most significant negatively enriched pathways after psymberin treatment, determined using gene set enrichment analysis. *Corresponds to Reactome, **Kegg, and ***Hallmark.

To better understand the mechanism of action for psymberin, we performed RNA-Seq on two low-passage CRC cell lines (CRC119 and CRC16-159) treated with psymberin. At the gene level, we observed substantial overlap in all mRNAs (non-coding and protein-coding mRNAs) (Figure 2A) and mRNAs of protein-coding genes (Figure 2B) for both CRC119 and CRC16-159 lines. At the pathway level, we observed a consistent positive enrichment in pathways related to differentiation, NF-κB signaling, and pathways relevant to tumor-immune cross-talk (IL-10 signaling, cytokine receptor interaction) (Figure 2C) and negative enrichment in multiple pathways, including eukaryotic protein elongation and ribosome pathways (Figure 2D).

**FIGURE 3:**
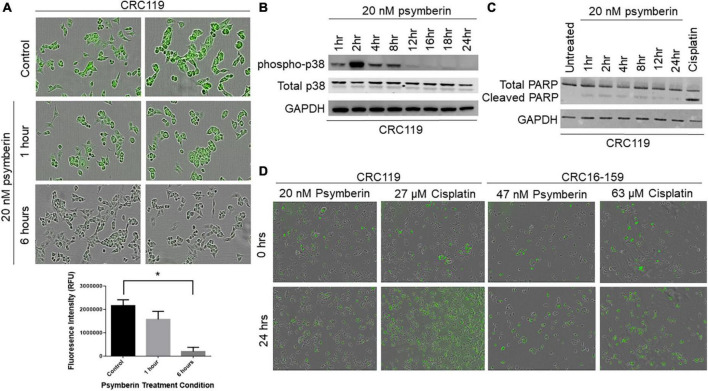
Psymberin induces protein synthesis inhibition and p38 activation. **(A)** Protein synthesis assay in CRC119 cells that were treated with 20 nM psymberin for one and 6 h (top). Quantification of fluorescence in protein synthesis assay (bottom) *p < 0.05. **(B)** Western blot for phospho-p38 in CRC119 cells treated with 20 nM psymberin at different time points. Total p38 and GAPDH are included as loading controls. **(C)** Western blot for cleaved PARP in CRC119 cells treated with 20 nM psymberin at different time points. GAPDH is included as a loading control. **(D)** Annexin V staining for protein translation in CRC119 and CRC16-159 at 0 and 24 h after treatment with psymberin.

The observation that psymberin inhibits mRNAs involved in translation and protein synthesis is consistent with previous studies suggesting translation inhibition as a proposed mechanism of action for psymberin (30, 31). To further confirm this, we used a fluorescent reporter of protein synthesis in which the incorporation of a methionine analog into newly synthesized proteins can be quantified by “click” chemistry (Thermo Fisher Scientific). Using this system, we observed rapid inhibition of protein synthesis as early as 1 h after treatment with psymberin, with nearly complete loss of signal by 6 h (Figure 3A).

Previous studies have shown a connection between translation inhibition and cellular stress pathways (32, 33), such as p38/MAPK activation. Consistent with these studies, we observed a rapid increase in levels of phospho-p38 upon treatment with psymberin, with the greatest increase at 2 h post treatment (Figure 3B). Cell cycle analysis on CRC401, CRC119, and CRC16-159 cells treated with psymberin indicated that psymberin led to significant G1 arrest in the cell line models and G2 arrest in the CRC404 organoid model (Supplementary Figure 2). Despite this protein translation inhibition and cell cycle arrest, however, analysis of apoptosis pathway markers by western blotting revealed low levels of cleaved PARP during treatment with psymberin (Figure 3C). Similarly, we observed no change in annexin uptake during treatment with psymberin for up to 24 h (Figure 3D). This is in contrast to cisplatin, which induced both cleaved caspase and increased annexin uptake. Together, these results suggest that psymberin inhibits protein synthesis, induces phosphorylation of p38, and leads to cell cycle arrest.

### Psymberin inhibits growth of colorectal cancer patient-derived organoids

**FIGURE 4:**
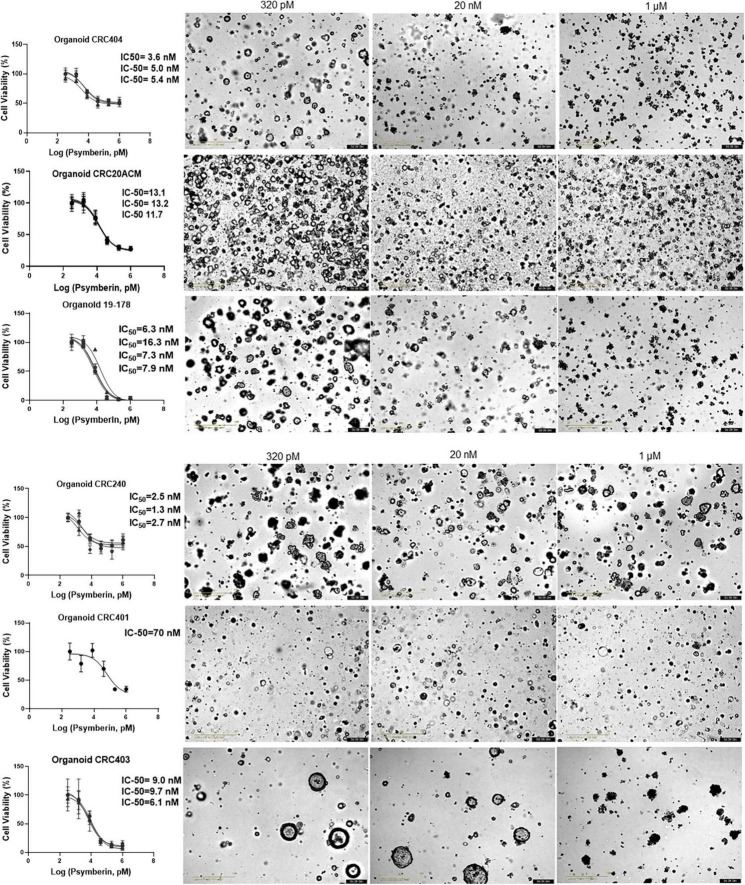
Psymberin has IC-50 values in the nanomolar level across multiple colorectal cancer (CRC) patient-derived organoids. Dose response curves are shown for six different CRC patient-derived organoids. Each experimental repeat is depicted in a different curve with different IC-50 values listed on the side of each curve. Images beside each graph show organoids from each line treated with 320 pM **(left)** and 1 μM **(right)** of psymberin.

To further validate the effectiveness of psymberin to induce CRC cell growth inhibition, we performed dose response assays with psymberin across a panel of six CRC patient-derived organoids (Figure 4). With the exception of one organoid line (CRC401; IC_50_ ∼ 70 nM), the IC_50_ values were all below 20 nM. Morphologically, organoids treated with a low dose of psymberin appear rounded, with refractile spheres throughout the culture. Conversely, organoids treated with higher doses (1 μM) of psymberin are dark and condensed, with few to no viable cells (Figure 4). Comparison of psymberin treatment in CRC240 grown as monolayer cell lines and organoids showed no difference in the IC_50_ values between the two growth conditions.

### Psymberin subunits do not inhibit the growth of colorectal cancer patient-derived organoids

**FIGURE 5:**
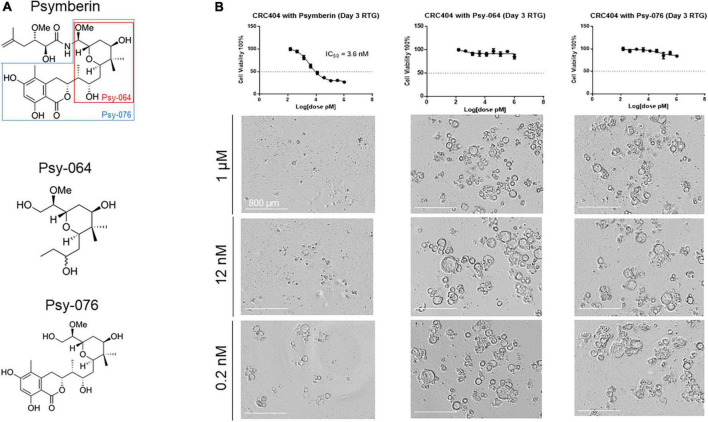
The entire structure of psymberin is important for its activity. **(A)** Structural comparison of psymberin and two of its analogs, Psy-064 and Psy-076. **(B)** Dose response curves for psymberin, Psy-064, and Psy-076 in CRC404 organoids. Images below each graph show organoids from each line treated with 1 μM (top) and 0.0002 μM (bottom) of that compound.

In addition to psymberin, we also quantified the cytotoxicity of two truncated psymberin analogs, Psy-064 and Psy-076 (Figure 5A). Both analogs are portions of the original psymberin compound, and Psy-064 itself is a component of Psy-076 (Figure 5A). To validate the effectiveness of the psymberin analogs, dose response assays were performed on CRC MicroOrganoSpheres using psymberin and both of its analogs. Consistent with our previous analyses, psymberin treatment of CRC MicroOrganoSpheres resulted in an IC_50_ ∼ 3.6 nM and visible inhibition of organoid growth, as noted by the reduction in size and collapse in spherical structure of the MicroOrganoSpheres (Figure 5B); however, the analogs had no negative impact on cell viability either in RealTime Glo fluorescence assays or observed visually (Figure 5B and Supplementary Figure 3). This suggests that the three-dimensional conformation of psymberin and the psymberic acid side chain may be required for its activity.

## Discussion

Patient-derived models of cancer, such as early-passage cell lines, PDXs, and organoids, have increasingly been accepted as “standard” preclinical models of cancer (8, 12–14). Each model has its own benefits and drawbacks. For example, early-passage cell lines are the cheapest to maintain of the three models (34). However, cells in monoculture are not representative of cancer growth in the human body and are not able to replicate the complexity of the tumor microenvironment (13, 35). Moreover, successful establishment of cell lines from patient tissue is extremely rare, regardless of cancer type (34). On the other hand, PDXs more closely model the tumor microenvironment (14) and tumor heterogeneity (14, 35). One major drawback to PDXs, however, is the high cost and time required to maintain these models (34). For these reasons, patient-derived organoids are growing in popularity because they are (1) able to model tumor heterogeneity (12, 13, 36); (2) have a higher uptake rate compared to cell lines (12, 34); (3) can be used to model the tumor microenvironment (36); and (4) are cheaper and faster to grow and maintain than PDXs (34).

We have previously used early passage cell lines and PDXs to develop a precision medicine pipeline to determine patient-specific targets for treatment (10). In this study, we utilize the latest version of our precision medicine pipeline, coupling cell lines and organoids to identify, test, and characterize a natural product library for potential anti-cancer compounds to treat CRC (Figure 1A). Using our pipeline, we identified psymberin as a potential anti-cancer agent for CRC (Figures 1B,C).

Psymberin, also known as irciniastatin A, was independently isolated by Pettit et al. and Cichewicz, Valeriote, and Crews in 2004 (15, 16). It was later confirmed by Jiang et al. (37) that the compounds isolated by both groups were, in fact, identical, despite being from two different types of sponges: *Psammocinia* sp. and *Ircinia ramosa* (15, 16). Both groups showed that psymberin was active against multiple cancer types, including CRC (15, 16). Moreover, multiple studies, including ours, have shown that psymberin is an extremely potent compound, with IC_50_ values in the low nanomolar range [Figures 1D, 4 and Supplementary Figure 1; (15, 16, 30, 38)].

The biological properties of psymberin have drawn considerable attention from research groups to develop a complete synthesis of the compound and identify its molecular mechanisms (18, 38, 39). Psymberin has previously been shown to inhibit translation in human leukemia (30) and lung carcinoma cells. This correlates with our data demonstrating that psymberin inhibits translation in CRC (Figures 2D, 3A). The inhibition of translation is connected to cellular stress pathways (32, 33). One such cell stress pathway involves p38/MAPK activation. Both Chinen et al. (30) and we have shown that psymberin induces p38 activation (Figure 3B).

Strong activation of p38 through phosphorylation has been associated with apoptosis, senescence, and terminal cell differentiation (40, 41). Our data suggest that by activating p38, psymberin may lead to G1 cell cycle arrest in CRC (Figures 3C,D and Supplementary Figure 2). While some studies have shown that psymberin induces apoptosis in other cancer types (30, 42), our results are consistent with previous studies linking p38 activation to G1 arrest (43–45).

Overall, psymberin is an extremely effective drug against CRC, both in cell line and organoid form, with IC_50_ values below 10 nM. Our results suggest that psymberin may inhibit protein translation in CRC and induces the upregulation of p38, leading to cell cycle arrest. Future studies should focus on evaluating toxicity and anti-tumor efficacy in *in vivo* settings.

## Data availability statement

The original contributions presented in the study are publicly available. This data can be found here: https://www.ncbi.nlm.nih.gov/bioproject/909790; project accession number: PRJNA909790.

## Ethics statement

The studies involving human participants were reviewed and approved by Duke IRB. The patients/participants provided their written informed consent to participate in this study.

## Author contributions

JS, JH, and DH: conceptualization, methodology, and supervision. DD, JM, GR, RR, and SV: formal analysis. IS, YZ, SK, and SM: resources. DD, JS, JH, and DH: writing—original draft. DD, JS, JM, GR, RR, SV, IS, YZ, SK, SM, JH, and DH: writing—review and editing. All authors contributed to the article and approved the submitted version.

## Supplementary material

Supplementary Figure 1 Psymberin has IC-50 values in the nanomolar level across multiple colorectal cancer cell lines. Dose response curves are shown for six different colorectal cancer cell lines. Each experimental repeat is depicted in a different curve with different IC-50 values listed on the side of each curve.

Supplementary Figure 2 Psymberin treatment leads to G1 cell cycle arrest in CRC cells. **(A)** Cell count for cells in different phases of cell cycle when untreated and treated with psymberin. The P5 label refers to G1, P6 refers to S phase, and P7 refers to G2. **(B)** Percent of cells in different phases of cell cycle in untreated and psymberin-treated cells. **(C)** Cell cycle analysis by flow cytometry in the CRC404 patient-derived organoid. **(D)** Quantification of cell cycle analysis based on the gates shown in panel **(C)**. **p* < 0.05 by chi-square test.

Supplementary Figure 3 The entire structure of psymberin is important for its activity. Dose response curves for psymberin, Psy-064, and Psy-076 in CRC420 organoids. Images below each graph show organoids from each line treated with 1 μM **(top)** and 0.0002 μM **(bottom)** of that compound.
