# Corrigendum: Psymberin, a marine-derived natural product, induces cancer cell growth arrest and protein translation inhibition

**DOI:** 10.3389/fmed.2023.1193745

**Published:** 2023-05-31

**Authors:** Divya L. Dayanidhi, Jason A. Somarelli, John B. Mantyh, Gabrielle Rupprecht, Roham Salman Roghani, Sophia Vincoff, Iljin Shin, Yiquan Zhao, So Young Kim, Shannon McCall, Jiyong Hong, David S. Hsu

**Affiliations:** ^1^Division of Medical Oncology, Department of Medicine, Duke University Medical Center, Durham, NC, United States; ^2^Center for Genomics and Computational Biology, Duke University, Durham, NC, United States; ^3^Department of Chemistry, Duke University, Durham, NC, United States; ^4^Department of Molecular Genetics and Microbiology, Duke University, Durham, NC, United States; ^5^Department of Pathology, Duke University, Durham, NC, United States; ^6^Department of Pharmacology and Cancer Biology, Duke University School of Medicine, Durham, NC, United States

**Keywords:** patient-derived organoids, patient-derived models of cancer, precision medicine, psymberin, high-throughput screening, protein translation

In the published article, there was an error in the Funding statement. A funding agency was not included in the Funding statement. The correct Funding statement appears below.

This research was partially supported by a grant of the Korea Health Technology R&D Project through the Korea Health Industry Development Institute (KHIDI), funded by the Ministry of Health and Welfare, Republic of Korea (grant number: HI19C1343).

The authors apologize for this error and state that this does not change the scientific conclusions of the article in any way. The original article has been updated.

