# Supplementary figures and images for: Psymberin, a marine-derived natural product, induces cancer cell growth arrest and protein translation inhibition
Source: Front Med (Lausanne). 2022 Dec 20;9:999004. doi: 10.3389/fmed.2022.999004 (PMC9894252; doi:10.3389/fmed.2022.999004)

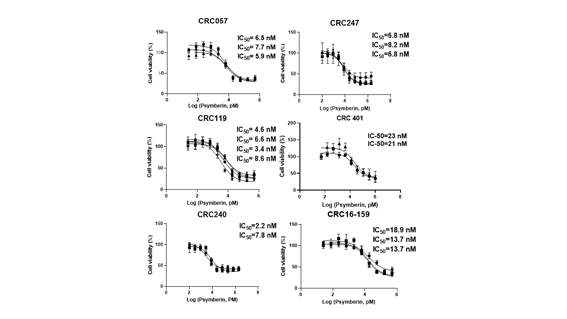

Supplement: Supplementary Figure 1 — Psymberin has IC-50 values in the nanomolar level across multiple colorectal cancer cell lines. Dose response curves are shown for six different colorectal cancer cell lines. Each experimental repeat is depicted in a different curve with different IC-50 values listed on the side of each curve. [file Image_1.TIF]

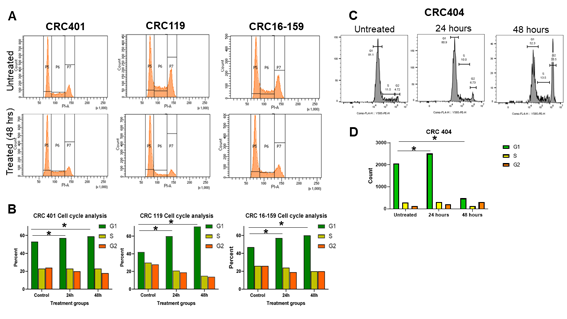

Supplement: Supplementary Figure 2 — Psymberin treatment leads to G1 cell cycle arrest in CRC cells. (A) Cell count for cells in different phases of cell cycle when untreated and treated with psymberin. The P5 label refers to G1, P6 refers to S phase, and P7 refers to G2. (B) Percent of cells in different phases of cell cycle in untreated and psymberin-treated cells. (C) Cell cycle analysis by flow cytometry in the CRC404 patient-derived organoid. (D) Quantification of cell cycle analysis based on the gates shown in panel (C). *p < 0.05 by chi-square test. [file Image_2.TIF]

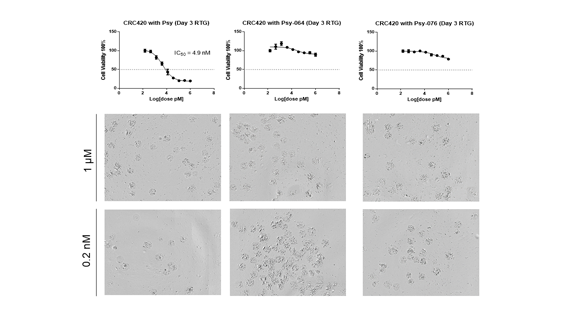

Supplement: Supplementary Figure 3 — The entire structure of psymberin is important for its activity. Dose response curves for psymberin, Psy-064, and Psy-076 in CRC420 organoids. Images below each graph show organoids from each line treated with 1 μM (top) and 0.0002 μM (bottom) of that compound. [file Image_3.TIF]
